# Emerging role of METTL3 in inflammatory diseases: mechanisms and therapeutic applications

**DOI:** 10.3389/fimmu.2023.1221609

**Published:** 2023-08-21

**Authors:** Bimei Song, Yue Zeng, Yanqing Cao, Jiamin Zhang, Chao Xu, Yaping Pan, Xida Zhao, Jingbo Liu

**Affiliations:** Department of Periodontics, School of Stomatology, China Medical University, Shenyang, China

**Keywords:** m6A modification, METTL3, inflammation, inflammatory diseases, immune cell, therapy

## Abstract

Despite improvements in modern medical therapies, inflammatory diseases, such as atherosclerosis, diabetes, non-alcoholic fatty liver, chronic kidney diseases, and autoimmune diseases have high incidence rates, still threaten human health, and represent a huge financial burden. N6-methyladenosine (m6A) modification of RNA contributes to the pathogenesis of various diseases. As the most widely discussed m6A methyltransferase, the pathogenic role of METTL3 in inflammatory diseases has become a research hotspot, but there has been no comprehensive review of the topic. Here, we summarize the expression changes, modified target genes, and pathogenesis related to METTL3 in cardiovascular, metabolic, degenerative, immune, and infectious diseases, as well as tumors. In addition to epithelial cells, endothelial cells, and fibroblasts, METTL3 also regulates the function of inflammation-related immune cells, including macrophages, neutrophils, dendritic cells, Th17 cells, and NK cells. Regarding therapeutic applications, METTL3 serves as a target for the treatment of inflammatory diseases with natural plant drug components, such as emodin, cinnamaldehyde, total flavonoids of *Abelmoschus manihot*, and resveratrol. This review focuses on recent advances in the initiation, development, and therapeutic application of METTL3 in inflammatory diseases. Knowledge of the specific regulatory mechanisms involving METTL3 can help to deepen understanding of inflammatory diseases and lay the foundation for the development of precisely targeted drugs to address inflammatory processes.

## 1 Introduction

Inflammation is a protective homeostatic response, characterized by the activation of multiple immune and non-immune cells to eliminate harmful stimuli and promote tissue repair (1–3). At the cellular and molecular level, inflammation can be understood as a sophisticated regulatory network, composed of inducers, sensors, mediators, and effectors (2). Inducers can be divided into exogenous and endogenous inducers, where exogenous inducers include microbes and microbial products, allergens, foreign bodies, and toxic compounds (4), and endogenous inducers are derived from stressed or dead cells and damaged or dysfunctional tissues (2). Inducers can be detected by transmembrane or cytoplasmic sensors, such as Toll-like receptors (TLRs), C-type lectin receptors, retinoic acid-inducible gene (RIG)-I-like receptors, and NOD-like receptors (NLRs) (5), and trigger inflammatory mediators, including vasoactive amines, vasoactive peptides, lipid mediators, cytokines, chemokines, and proteolytic enzymes (2). These inflammatory mediators eventually affect the functional activities of effectors, including monocytes, macrophages, neutrophils, dendritic cells (DCs), lymphocytes, and mast cells, which are immune cells, as well as non-immune epithelial cells, fibroblasts, and endothelial cells (6).

Timely shutdown of inflammation is crucial for physiological homeostasis (6), and dysregulation of this process leads to chronic inflammation (3, 7). Although the regulatory mechanisms underlying chronic inflammation are not completely clear, it is established as associated with aging, chronic infection, unhealthy lifestyle, diet, and adverse environmental conditions (8). At the molecular level, chronic inflammation is usually triggered by endogenous danger signals (9) and causes tissue dysregulation, subsequently leading to various chronic inflammatory diseases, including cardiovascular diseases, type 2 diabetes, non-alcoholic fatty liver, rheumatoid arthritis, and neurodegenerative diseases, among others (3, 8, 10).

Recent studies indicate that RNA modification of N6-methyladenosine (m6A) is critical for inflammation (11); m6A describes methylation of internal adenosine residues in mRNA and is the most prevalent internal post-transcriptional modification of eukaryotic mRNA (12). m6A modification preferentially occurs at the consensus sequence, RRACH (R: purine, A: m6A site, H: non-guanine base), in mRNA (13, 14), and is preferentially located around stop codons, and enriched at 3’ untranslated regions and within long internal exons in human transcriptomes (15). The m6A methylation process is reversible and dynamically regulated by methyltransferases (writers), demethylases (erasers), and m6A-binding proteins (readers) (16). Methyltransferases (writers) add m6A to mRNA, and include METTL3 (17, 18), METTL14 (18), METTL16 (19), WTAP (20), KIAA1429 (21), RBM15 (22), and ZC3H13 (23). Further, m6A modification is removed by demethylases (erasers), including FTO (24) and ALKBH5 (25), while m6A-modified transcripts are recognized by m6A-binding proteins (readers), such as YTHDC1/2 (26, 27), YTHDF1/2/3 (28–30), HNRNPA2B1 (31), HNRNPC (32), and IGF2BP1/2/3 (33). Readers can bind to the m6A motif and affect mRNA metabolism, including mRNA splicing and export from the nucleus, stability, translation, and localization in the cytoplasm, thereby regulating gene expression at the post-transcriptional level (16).

METTL3 was the first m6A methyltransferase to be discovered. In 1997, Boker et al. isolated, purified, and sequenced METTL3 (also referred to as MT-A70) from HeLa cells, and confirmed that it is an important (N6-adenosine)-methyltransferase subunit (17). Together, METTL3, METTL14, and WTAP form the m6A methyltransferase complex (34), in which METTL3 is the core component that provides catalytic activity, while METTL14 functions as an RNA binding platform and WTAP is responsible for recruiting the complex to RNA targets (20, 35, 36). METTL3 functions in various pathophysiological processes, including the cell cycle, apoptosis, innate immunity, and inflammation, as well as cell proliferation, migration, invasion, and differentiation (37). As the m6A methylase most widely discussed in the context of inflammation and inflammatory diseases, the role of METTL3 in inflammatory diseases has not been systematically elucidated.

In this review, we describe the role of METTL3 in macrophages, neutrophils, DCs, T helper (Th)17 cells, and natural killer (NK) cells which play crucial pathological roles in chronic inflammation. We comprehensively summarize METTL3 expression changes and related pathogenesis in inflammatory diseases, including atherosclerosis, myocardial infarction (MI), non-alcoholic liver disease, hyperuricemia (HUA), diabetes nephropathy, osteoarthritis (OA), intervertebral disc degeneration (IDD), rheumatoid arthritis, psoriasis, inflammatory bowel disease (IBD), sepsis, pneumonia, and tumor. In conclusion, we focus on the therapeutic application of METTL3 in inflammatory diseases. Knowledge of the mechanisms by which METTL3 regulates inflammatory diseases will contribute to the development of improved targeted therapies and broaden clinical perspectives.

## 2 METTL3 regulates inflammation-related immune cell functions

### 2.1 METTL3 regulates macrophage function

Inflammation involves multiple immune cells, among which macrophages are key participants. Macrophages/monocytes are derived from progenitor cells in the bone marrow and enter the bloodstream (38). After being recruited, these cells can enter tissue through the vascular endothelium and become tissue-resident macrophages. Macrophages are involved in all aspects of the physiological and pathological processes of inflammation, including the perception of stimuli, phagocytosis of pathogens, removal of cell debris, presentation of antigens, secretion of cytokines, and promotion of tissue repair.

Macrophages are plastic and, when stimulated by the microenvironment, can polarize into different phenotypes, which are generally divided into classic activated or inflammatory (M1) and alternate activated or anti-inflammatory (M2) macrophages (39). M1 macrophages are activated by IFN-γ and TNF-α, or by lipopolysaccharide (LPS). They secret proinflammatory cytokines, such as TNF-α, IL-1α, IL1-β, IL-6, IL-12, IL-23, nitric oxide (NO), and cyclooxygenase-2 and produce reactive oxygen species (ROS). Functionally, M1 macrophages can remove pathogens and can also contribute to tissue damage (40). M2 macrophages are activated by IL-4, IL-13, and IL-10, and secrete IL-10 and TGF-β. M2 macrophages can clear apoptotic cells, promote angiogenesis and fibrosis, and coordinate tissue remodeling (41). Macrophages play a defensive role in acute inflammation but have strong pathological effects in chronic inflammation and inflammatory diseases (42, 43), and macrophage polarization is the pathological basis of many chronic diseases driven by inflammation (39, 40).

**Figure 1:**
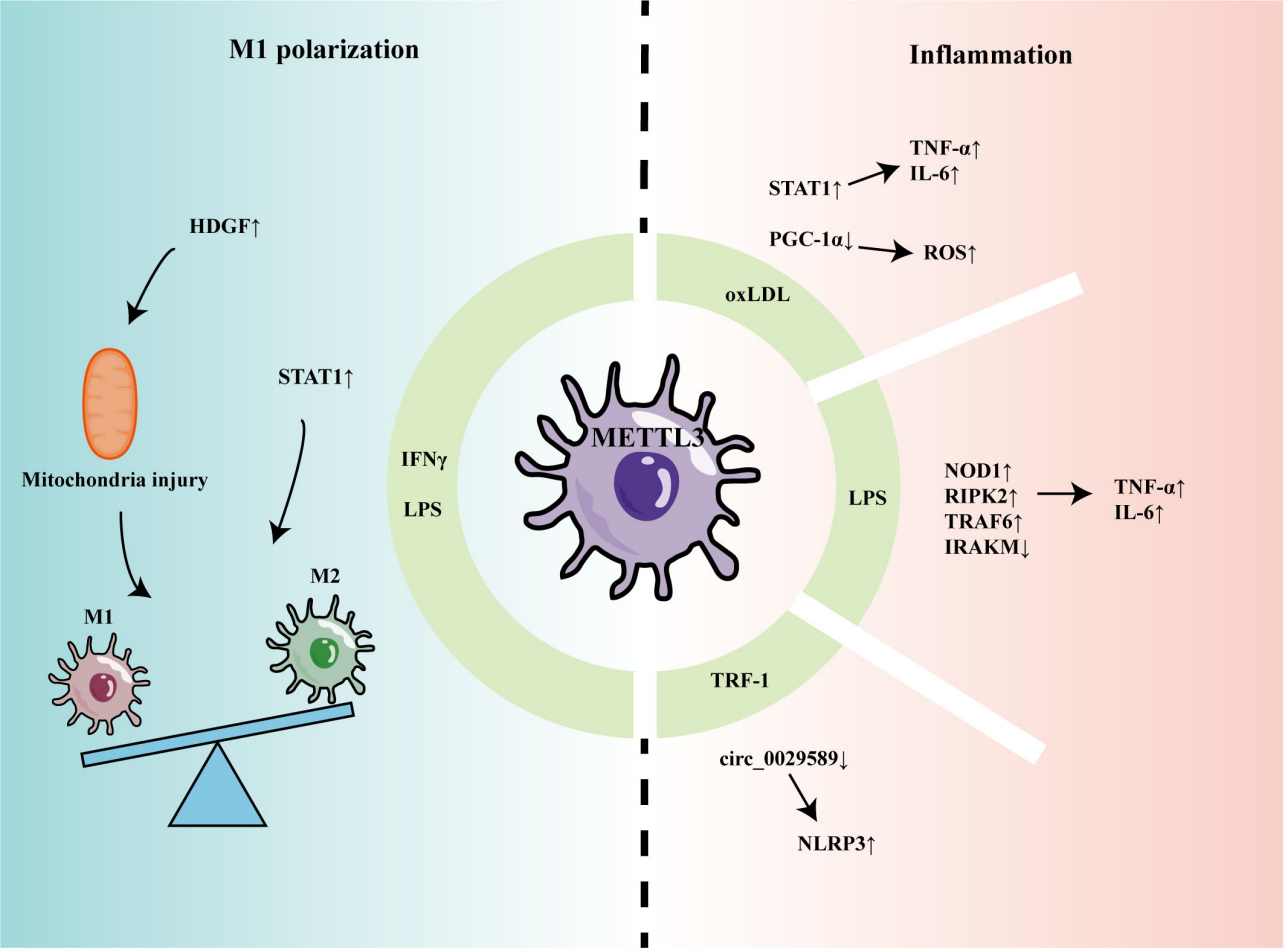
The role of METTL3 in regulating macrophage inflammation and polarization.

Recent studies have indicated that METTL3 can participate in regulating macrophage-mediated inflammation and M1 polarization (**Figure 1**). Li et al. found that m6A methylation and METTL3 levels were increased in macrophages stimulated by oxidized low-density lipoprotein (oxLDL) (44). Mechanistically, METTL3 promoted signal transducer and activator of transcription 1 (STAT1) expression by increasing the m6A level of the *STAT1* transcript in macrophages, thereby elevating the levels of downstream inflammatory factors (44). In another study, Sun et al. found that, although oxLDL could mildly promote expression levels of METTL3 and METTL14, it decreased interaction between METTL3 and METTL14, leading to a reduction in overall m6A levels in macrophages (45). Further, Sun et al. found that Matrin-3 was reduced in macrophages stimulated by oxLDL, and involved in the formation of the METTL3-METTL14 complex, thus affecting its m6A modification of peroxisome proliferator-activated receptor gamma coactivator 1-alpha (*PGC-1α*) mRNA (45). METTL3 was proven to co-modify *PGC-1* mRNA with YTHDF2 and inhibit PGC-1 expression, which reduced ATP production and increased ROS accumulation, eventually aggravating inflammatory responses (46). Guo found that *circ_0029589* expression was down-regulated in peripheral blood mononuclear cell-derived macrophages from patients with acute coronary syndrome and that IRF-1 inhibited *circ_0029589* through METTL3-mediated m6A modification, thereby facilitating macrophage pyroptosis and inflammation (47).

In another report of macrophage stimulation using LPS, levels of METTL3 and m6A modification were decreased (48). METTL3 knockdown promoted the stability of nucleotide-binding oligomerization domain containing 1 (*NOD1*) and receptor-interacting serine/threonine kinase 2 (*RIPK2*) mRNA, mediated by YTHDF2, thereby up-regulating levels of TNF-α, IL-6, and NO (48); however, Tong et al. found that METTL3-deficient macrophages produced less TNF-α and that METTL3 knockout mice had higher bacterial loads in the feces and cecum, indicating that METTL3 promotes the anti-infection activity of macrophages (49). Mechanistically, METTL3 deficiency reduced *Irakm* (a negative regulatory factor in the TLR4 signaling pathway) mRNA degradation and promoted its expression. Microglia are often referred to as tissue-resident macrophages of the central nervous system (50). When damage or infection is detected, microglia can transform into an activated phenotype with inflammatory functions, which is important in a variety of neuropathological conditions. METTL3 was increased in LPS-stimulated microglia and promoted TRAF6/NF-κB pathway expression by m6A modification of *TRAF6*, thereby inducing expression of inflammatory cytokines, including IL-1β, IL-6, TNF-α, and IL-18 (51).

METTL3 also promotes proinflammatory M1 polarization. METTL3 was increased in mouse bone marrow-derived M1 macrophages and methylated the mRNA encoding STAT1, a transcription factor that regulated M1 macrophage polarization, promoting its expression (52). Further, METTL3 promoted M1 polarization by regulating macrophage metabolism. METTL3 could upregulate hepatoma-derived growth factor (HDGF) expression in macrophages by enhancing its mRNA stability (53). Pro-inflammatory M1 macrophages mainly rely on the glycolysis pathway to generate energy (54), while HDGF promotes M1 macrophage polarization by inducing mitochondrial injury and thereby stimulating glycolysis (53).

### 2.2 METTL3 regulates neutrophil activation

Neutrophils are the first innate immune cells recruited to sites of infection. The anti-infection ability of neutrophils depends on migration, phagocytosis, degranulation, and formation of neutrophil extracellular traps (NETs). Neutrophils were considered to mainly be involved in acute inflammation, but it is now widely believed that neutrophils also have important roles in cancer, autoimmune diseases, and chronic inflammation (55). METTL3 levels were increased in bone marrow neutrophils from LPS-treated C57BL/6 mice and affected neutrophil migration, while METTL3 knockout reduced the release of both bone marrow and circulating neutrophils (56). Mechanistically, METTL3 mediated m6A modification of *TLR4* mRNA, thus enhancing its expression and stimulating TLR4 pathway activation, leading to increased cytokine production and promoting CXCR2 expression (56).

### 2.3 METTL3 regulates DC activation

DCs are heterogeneous antigen-presenting cells that connect innate and adaptive immunity. DCs mediate immune defense and immune tolerance and disordered DC migration or activation can have pathological consequences associated with autoimmune and infectious diseases (57). METTL3 is involved in regulating DC activation. m6A modification and METTL3 levels were increased in mature bone marrow DCs from C57BL/6 mice (58), while METTL3 knockout could inhibit DC maturation and cytokine production (58, 59). METTL3 increased *CD40* and *CD80* mRNA m6A modification levels, thereby promoting CD40 and CD80 expression (58); these two membrane co-stimulatory molecules assist in antigen presentation and T-cell activation. In addition, METTL3 promoted *Tirap* expression by increasing its m6A modification level, which enhanced the activity of the TLR4/NF-κB signaling pathway and cytokine secretion (58).

### 2.4 METTL3 regulates Th17 cell function

Among Th cell subsets, Th17 cells are considered inflammatory, and their differentiation and pathogenic functions are related to various inflammatory and autoimmune diseases, such as psoriasis and rheumatoid arthritis (60). Particular cytokines and environmental stimuli induce Th17 cell differentiation and secretion of the pathogenic cytokine, IL-17 (61, 62). METTL3 was recently identified as involved in regulating the pathogenic function of Th17 cells. METTL3 and m6A modification were reduced in T cells from mice with experimental autoimmune uveitis (63), and METTL3 overexpression inhibited the differentiation of Th17 phenotype cells and reduced IL-17 secretion (63). Mechanistically, METTL3 increased *ASH1L* mRNA stability, which negatively regulates Th17 cell generation and function (63). In addition, elevated METTL3 and m6A modifications were found in bone samples from patients with periprosthetic joint infection (64); KEGG analysis demonstrated that differentially methylated genes are mainly enriched in Th17 cell differentiation and the IL-17 signaling pathway, indicating that Th17 cells and METTL3 may contribute to the pathogenesis of periprosthetic joint inflammation (64).

### 2.5 METTL3 regulates NK cell function

NK cells play an important role in virus and tumor immunity. NK cells can sense infected and tumor cells through natural cytotoxicity receptors, C-type lectin-like receptors, and co-activating receptors (65). Upon activation, NK cells release lytic granules and cytokines, and collaborate with other immune cells to kill target cells (66). In addition, NK cells can also activate death ligands, which induce target cell apoptosis (67). Recently, METTL3 has been reported to be associated with the function of infiltrating NK cells in tumors. Song et al. found that METTL3 was decreased in NK cells from patients with hepatocellular carcinoma (68). METTL3 knockdown inhibited NK cell infiltration and reactivity to IL-15, which is related to suppressed activation of AKT and MAPK pathways (68).

## 3 The role of METTL3 in inflammatory diseases

**Figure 2:**
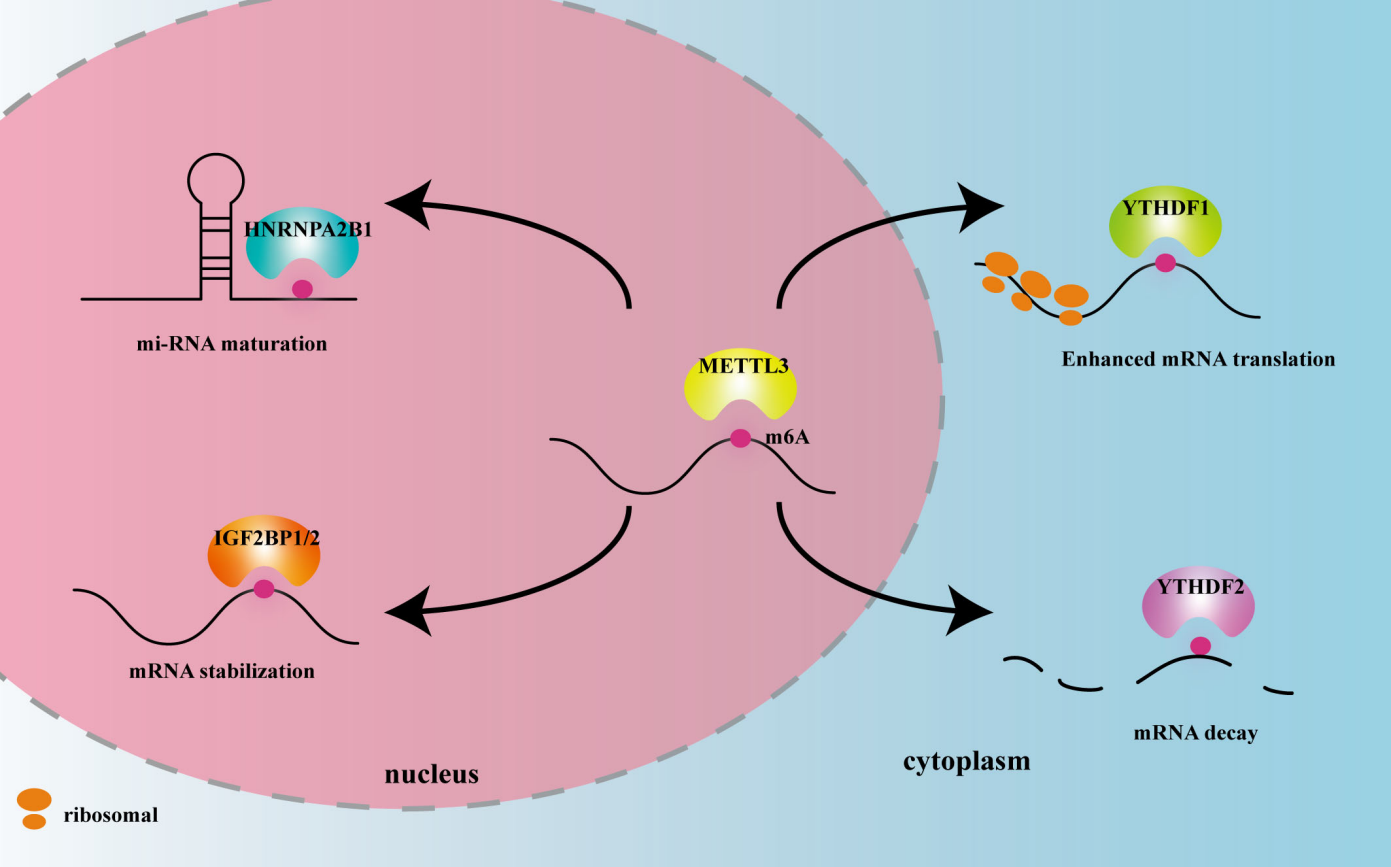
The effects of METTL3 and m6A reader proteins mentioned in this review on RNA modification and metabolism. Figure Legends: METTL3 adds the m6A motif to RNA. HNRNPA2B1 recognizes m6A in the nucleus and promotes miRNA maturation. IGF2BP1/2 recognizes m6A in the nucleus and promotes mRNA stability. YTHDF1 recognizes m6A in the cytoplasm and enhances mRNA translation. YTHDF2 recognizes m6A in the cytoplasm and promotes mRNA decay.

METTL3 plays important roles in both inflammation-related immune cells and other cell types, such as epithelial cells, endothelial cells, and fibroblasts. METTL3 expression levels can change under disease conditions, influencing expression of downstream target genes, and thereby participating in inflammatory disease pathogenesis. In the following sections, we elaborate on the mechanism of action of METTL3 and its effects on m6A reading proteins in cardiovascular, metabolic, degenerative, immune, infectious diseases, and tumors. The effects of METTL3 and the m6A reading proteins discussed in this review on RNA modification and metabolism are summarized in **Figure 2**. Expression changes and pathogenic mechanisms of METTL3 in various inflammatory diseases are shown in **Tables 1**, **2**.

**Table 1:** Changes of METTL3 expression in inflammatory diseases.

Diseases	In patient	*In vivo*	*In vitro*	Ref
Atherosclerosis	–	Decreased in AS lesions of sh-METTL3 mice	Increased in ox-LDL-induced human umbilical vein endothelial cells	(69)
	–	Decreased in AS regions of mice	Decrease in OS-stimulated human umbilical vein endothelial cells	(70)
	–	Increased in AS regions of mice	Increased in OS-stimulated human umbilical vein endothelial cells and mouse aortic endothelial cells	(71)
	–	–	Increased in IFNγ and LPS-induced macrophage	(53)
Acute coronary syndrome	Increased in peripheral blood mononuclear cells from ACS patients	–	–	(47)
Myocardial infarction	–	Increased in rat MI model	–	(72)
	–	Increased in rat MI model	–	(73)
Non-alcoholic fatty liver disease	Increased in liver tissues from NASH patients (GSE89632)	Increased in macrophages derived from obese leptin-deficient mice (GSE54154)	–	(74)
Increased in nuclei of NASH patients livers	Increased in leptin receptor deficiency mice livers	–	(75)
	–	Increased in Kupffer cells from *in vivo* liver fibrosis model	Increased in bone-marrow-derived macrophages and RAW264.7	(76)
	Hyperuricemia	–	Decreased in HUA mice’s hippocampus	–	(77)
Acute Kidney Injury	Increased in kidney from patients with AKI	Increased in AKI mouse model	Increased in tubular epithelial cells	(78)
Diabetic nephropathy	Increased in kidney of patients with DN	Increased in kidney of DN mice	–	(79)
	–	–	Decreased in high glucose-induced podocytes	(80)
Renal fibrosis	–	Increased in obstructed kidney tissue of mice	–	(81)
Osteoarthritis	–	Increased in cartilage tissue of OA mouse model	Increased in IL-1β-treated mouse chondrocyte line (ATDC5)	(82)
	Decreased in articular cartilages of OA patients	–	Decreased in IL-1β-treated chondrosarcoma (SW1353) cells	(83)
	–	Decreased in cartilage of TMJ OA mice	Decreased in TNF-α- stimulated chondrocytes (ATDC5)	(84)
	–	Increased in cartilage tissues of OA mice	–	(85)
	Increased in FLS isolated from the synovium of patients with OA	Increased in DMM-induced OA mice models	–	(86)
Intervertebral disc degeneration	Increased in NPCs from IDD patients	–	–	(87)
Rheumatoid arthritis	Increased in the PBMCs of patients with RA	–	Increased in LPS-induced THP-1 macrophages	(88)
	Increased in human RA synovial tissues	Increased in RA rat model	–	(89)
Psoriasis	Decreased in psoriatic skin lesions of patients	–	–	(90)
Inflammatory bowel diseases	Increased in IBD patients	Increased DSS-induced IBD mice	Increased in LPS-treated MODE-K cells	(91)
Sepsis	Decreased in the aortic tissue of LPS-induced sepsis rat model	–	–	(92)
Sepsis-associated ARDS	–	–	Increased in NETs-treated alveolar epithelial cells	(93)
	–	Increased in lung tissues of SI-ALI mice group	Increased in NETs-alveolar epithelial cells	(94)
	–	Decreased in LPS-induced ARDs mice	Decreased in LPS-induced HULEC-5a	(95)
	–	Increased in ARDS mice	–	(96)
Pediatric pneumonia	Increased in peripheral blood monocytes from pediatric pneumonia patients	–	Increased in WI-38 cells	(97)
Neonatal pneumonia	Increased in NP patients	–	Increased in LPS-induced WI-38 cells	(98)
SARS-CoV-2 infection	Decreased in severe COVID-19 patients	–	–	(99)
Fungal keratitis	–	Increased in corneas of FK mouse model	Increased in fungi-infected corneal stromal cells	(100)
	–	Increased in corneas of FK mouse model	Increased in fungi-infected corneal stromal cells	(101)
Intraocular inflammatory disease	–	–	Increased in LPS-induced RPE cells	(102)
Pulpitis	–	–	Increased in LPS-induced human dental pulp cells	(103)
B16 tumors	–	–	Decreased in TAMs and BMDMs	(104)
Papillary thyroid cancer	Decreased in papillary thyroid cancer tissue	–	Decreased in papillary thyroid cancer tissue cell	(105)
Prostate cancer	–	–	Decreased in prostate cancer macrophages	(106)
Lung adenocarcinoma	–	–	Increased in human lung cancer A549 and H1975 cell lines	(107)
Colorectal cancer	Increased in colorectal cancer tissue from oxaliplatin-resistant patients	–	–	(108)

**Table 2:** METTL3-associated pathogenesis in inflammatory diseases.

Diseases	Target	Reader	Mechanism	Ref
Atherosclerosis	JAK2↑	IGF2BP1	METTL3 depletion reduced the stability of JAK2 mRNA and suppressed JAK2/STAT3 pathway	(69)
	EGFR↓	–	METTL3 depletion slowed down EGFR mRNA decay and activated TSP-1/EGFR axis	(70)
	NLRP1↑;KLF4↓	–	METTL3 methylation of NLRP1 and KLF4 mRNA led to stabilization of NLRP1 mRNA and the degradation of KLF4 mRNA. NLRP1 promoted epithelial inflammation while KLF4 maintained vascular homeostasis	(71)
	HDGF↑	–	METTL3 knockdown decreased HDGF mRNA stability, thereby reducing aerobic glycolysis and lipids accumulation	(53)
Acute coronary syndrome	–	–	METTL3 knockdown decreased the m6A level of circ_0029589 which was required for macrophage pyroptosis	(47)
Myocardial infarction	TLR4	–	METTL3 knockdown decreased the expression of TLR4 and suppressed TLR4/NF-κB pathway and inflammatory cytokines production	(72)
	TRAF6↑	–	METTL3 knockdown reduced expression of TRAF6 and inhibited TRAF6/NF-κB pathway and ROS production	(73)
Non-alcoholic fatty liver disease	DDIT4↓	–	METTL3 knockout increased expression of DDIT4 which could inhibited the mammalian target of rapamycin C1 activity and NF-κB pathway	(74)
	CD36↓;CCL2↓	–	Hepatocyte-specific deletion of METTL3 promoting CD36-mediated hepatic free fatty acid uptake and CCL2-induced inflammation	(75)
	MALAT1↑	–	METTL3 upregulated MALAT1 and promoted pyroptosis and inflammation of macrophages through MALAT1/PTBP1/USP8/TAK1 axis	(76)
Hyperuricemia	–	–	Overexpressing METTL3 promoted maturation of miR-124-3p, which inhibited MyD88-NF-κB and NLRP3-ASC-Caspase1 inflammasome	(77)
Acute Kidney Injury	TAB3↑	IGF2BP2	METTL3 targeted TAB3 and its mRNA stability was increased through binding of IGF2BP2 and thus promoting TECs inflammation	(78)
Diabetic nephropathy	TIMP2↑	IGF2BP2	METTL3 knockout reduced m6A level of TIMP2 and downregulated its expression, which significantly reduced the inflammation and apoptosis via Notch pathway	(79)
	PTEN↓	–	METTL3 knockdown increased the expression of PTEN which promoted podocytes pyroptosis through PI3K/AKT signaling pathway	(80)
Renal fibrosis	pr-miR-21↑	HNRNPA2B1	Overexpressing METTL3 promoted miR-21-5p maturation and activated SPRY1/ERK/NF-κB pathway	(81)
Osteoarthritis	–	–	Silence of METTL3 reduced cells apoptosis and inhibited inflammatory response and extracellular matrix synthesis	(82)
	–	–	METTL3 overexpression reduced inflammatory cytokines and increased p65 and p-ERK protein	(83)
	Bcl2↑	YTHDF1	METTL3 overexpression increased Bcl2 mRNA through YTHDF1-mediated m6A modification and inhibited the apoptosis and autophagy	(84)
	SOCS2↓	–	RPL38 could bind with METTL3 and RPL38 knockdown increased expression of SOCS2 and thus alleviated chondrocyte apoptosis inflammation and ECM degradation	(85)
	ATG7↓	YTHDF2	METTL3 knockdown upregulated the expression of ATG7 in a YTHDF2-dependent manner and suppressed the senescence of FLSs	(86)
Intervertebral disc degeneration	SIAH1↑	–	METTL3 depletion reduced the stability of SIAH1 mRNA which induce apoptosis, inflammation, senescence, and decreased ECM synthesis in NPCs	(87)
Rheumatoid arthritis	–	–	METTL3 overexpression inhibited the proliferation of macrophages and NF-κB pathway	(88)
	–	–	METTL3 knockdown suppressed interleukin (IL)-6, matrix metalloproteinase (MMP)-3, and MMP-9 levels through inhibiting NF-κB pathway	(89)
Inflammatory bowel diseases	–	–	knockdown of METTL3 reduced levels of proinflammatory cytokines and inflammatory enzymes and inhibited phosphorylation of p65	(91)
Sepsis-associated ARDS	GPX4↓	YTHDF2	METTL3 methylated GPX4 mRNA and decreased GPX4 expression via YTHDF2-mediated degradation of GPX4mRNA, and thus lead to ferroptosis of alveolar epithelial cells	(93)
	Sirt1↓	–	METTL3 mediated m6A methylation of Sirt1 mRNA in alveolar epithelial cells, resulting in abnormal autophagy of cells	(94)
	Trim59↑	–	METTL3 knockdown reducing stability of Trim59 mRNA and activating NF-κB pathway	(95)
	circN4bp1	–	METTL3 inhibition reduced the expression of circN4bp1 which promoted M1 macrophage polarization	(96)
Pediatric pneumonia	–	–	METTL3 knockdown inhibited inflammatory response and cell apoptosis by reducing the expression of EZH2 and JAK/STAT3 pathway	(97)
Neonatal pneumonia	STAT2↑	YTHDF1	METTL3 knockdown reduced the translation of STAT2 mRNA mediated by YTHDF1 and alleviated LPS‐induced inflammatory damage	(98)
Rotavirus infection	IRF7↓	–	METTL3 knockout in intestinal epithelial cells increased IRF7 expression, which leads to the upregulation of IFNs and ISGs	(109)
SARS-CoV-2 infection	SARS-CoV-2 RNA↑	–	METTL3 depletion of host cell decreased SARS-CoV-2 viral load and enhanced RIG-I binding to viral RNA and phosphorylation of IRF3 and IκBα	(99)
Fungal keratitis	–	–	METTL3 knockdown attenuated the inflammatory response of FK through inhibiting PI3K/AKT pathway	(100)
	–	–	METTL3 knockdown attenuated the inflammatory response of FK through inhibiting TRAF6/NF-κB pathway	(101)
Intraocular inflammatory disease	NR2F1↑	–	Silencing METTL3 increased the expression of NR2F1 and promotes IL-6 secretion	(102)
Pulpitis	–	–	METTL3 knockdown reduced inflammatory cytokines secretion through NF-κB and MAPK pathway	(103)
Melanoma	SPRED2↑	–	METTL3 knockout inhibited the translation of SPRED2, thereby inhibiting SPRED2-mediated activation of NF- κB and STAT3 pathway	(104)
Glioma	SOCS2↓	–	METTL3 promoted SOCS2 degradation in glioma by promoting m6A modification of SOCS2	(110)
Prostate cancer	–	–	METTL3 knockdown promoted STAT6 phosphorylation	(106)
Papillary thyroid cancer	c-Rel↓	YTHDF2	METTL3 knockdown increased c-Rel and promoted NF-κB pathway, thereby increasing IL-8 and infiltration of neutrophils	(105)

↑up-regulated by METTL3; ↓down-regulated by METTL3

JAK2, Janus kinase 2; STAT3, signal transducer and activator of transcription 3;EGFR, epidermal growth factor receptor; TSP-1, thrombospondin-1; HDGF,hepatoma-derived growth factor; TLR4, toll-like receptor 4; TRAF6, TNF receptor associated factor 6; DDIT4, DNA damage inducible transcript 4; CCL2, C-C motif chemokine ligand 2; MALAT1, metastasis associated lung adenocarcinoma transcript 1 ; TAB3, TGF-beta activated kinase 1 (MAP3K7) binding protein 3; TIMP2, TIMP metallopeptidase inhibitor 2; PTEN, phosphate and tension homology; BCL2, BCL2 apoptosis regulator; SOCS2, suppressor of cytokine signaling 2; ATG7, autophagy related 7; SIAH1, siah E3 ubiquitin protein ligase 1; GPX4, glutathione peroxidase 4; Sirt1;sirtuin 1; Trim59, tripartite motif containing 59; STAT2, signal transducer and activator of transcription 2; IRF7, interferon regulatory factor 7; NR2F1, nuclear receptor subfamily 2 group F member 1; SPRED2, sprouty related EVH1 domain containing 2; c-Rel, REL proto-oncogene

### 3.1 Cardiovascular disease

#### 3.1.1 Atherosclerosis

Atherosclerosis is an immunoinflammatory disease featuring the formation of lipid plaques in the intima of arteries, resulting in lumen stenosis (111). The process of atherosclerosis development includes the proliferation of smooth muscle cells and monocytes, increased synthesis and secretion of collagen fibers by smooth muscle cells, and lipid accumulation. Dong et al. found that METTL3 was up-regulated in ox-LDL-induced human umbilical vein endothelial cells and METTL3 knockdown prevented the development of atherosclerosis (69). Functionally, METTL3 m6A modifies *JAK2* mRNA and IGF2BP1 enhances *JAK2* mRNA stability, while METTL3 knockdown inhibits cell proliferation and angiogenesis by inhibiting the JAK2/STAT3 pathway (69). Stenosis caused by an atherosclerosis plaque will affect blood flow and generate oscillatory stress (OS) in the plaque area. OS causes epithelial activation and deterioration of atherosclerosis. Li et al. found that METTL3 expression was decreased in atherosclerotic regions with OS, leading to reduced m6A modification of epidermal growth factor receptor (*EGFR*), causing slower *EGFR* mRNA decay and increased EGFR expression (70). EGFR is associated with atherosclerosis progression (70); however, Chien et al. found that METTL3 was up-regulated under OS (71). METTL3 methylation stabilizes *NLRP1* and leads to the degradation of *KLF4* mRNA, where NLRP1 promotes epithelial inflammation and KLF4 maintains vascular homeostasis (71). Zheng et al. explored the pathogenesis of atherosclerosis from the perspective of macrophage polarization and energy metabolism (53) and found that HDGF was highly expressed in the aortas of patients with atherosclerosis and APOE knockout mice, as well as in M1 macrophages. HDGF knockdown alleviated inflammation, glycolysis, and lipid accumulation in M1 macrophages, while high METTL3 expression in macrophages increased HDGF levels through m6A modification, thereby aggravating atherosclerosis.

#### 3.1.2 Myocardial infarction

Microglia inflammation in the paraventricular nucleus after MI results in excessive activation of the sympathetic nerve, leading to ventricular arrhythmia in patients with MI. Patients with MI have high mortality rates from sudden cardiac death caused by ventricular arrhythmia. Increased METTL3 elevates m6A modification of *TLR4* and up-regulates its expression, which promotes sympathetic hyperactivity via the TLR4/NF-κB pathway and increases the incidence of ventricular arrhythmias post-MI (72). In another study, Qi et al. found that METTL3 expression increased in rats after MI, and METTL3 targeted TRAF6 and promoted inflammation after MI, through the TRAF6/NF-κB pathway (73).

### 3.2 Metabolic disease

#### 3.2.1 Non-alcoholic fatty liver disease

Nonalcoholic fatty liver disease (NAFLD) describes conditions ranging from steatosis and non-alcoholic steatohepatitis (NASH), which is characterized by lobular inflammation and hepatocellular injury, through to cirrhosis and hepatocellular carcinoma, as the disease progresses (112). Liver-specific METTL3 knockout mice exhibit pathological features associated with NAFLD (113). Yet, in another study, myeloid lineage-restricted deletion of METTL3 protected against NAFLD development (74). The liver of myeloid-specific METTL3 knockout mice showed lower levels of lipid accumulation and monocyte infiltration, as well as low NAFLD activity inflammation scores (74). METTL3 appears to hinder NASH development. Li et al. showed that METTL3 overexpression alleviated NASH by suppressing the expression of CD36 and C-C motif chemokine ligand 2 (CCL2) (75); however, METTL3 regulated *CD36* and *CCL2* expression at the transcriptional level, rather than through m6A modification. METTL3 inhibited *CD36* and *CCL2* transcription by recruiting histone deacetylase 1/2 to induce deacetylation of H3K9 and H3K27 in the *CD36* and *CCL2* promoter regions. In contrast, in the liver fibrosis stage of NAFLD, METTL3 may become a promoter of disease. Shu et al. found that METTL3 was up-regulated in a liver fibrosis model and in macrophages, and aggravated liver fibrosis by stimulating macrophage pyroptosis and inflammation (76).

#### 3.2.2 Hyperuricemia

HUA is a metabolic disease caused by raised serum uric acid (> 6.5–7 mg/dL) (114). Epidemiological evidence indicates that HUA is related to various cardiovascular diseases (115) and cognitive impairment (116). In an *in vivo* experiment, levels of inflammatory cytokines in the hippocampus of rats in the HUA group were significantly higher than those in the negative control group (117). Further, in a clinical study, serum proinflammatory cytokines and oxidative stress were increased in asymptomatic young patients with primary HUA, who were in a chronic inflammatory state, which may be the reason underlying systemic disease (118). The latest evidence suggests that METTL3 is a bridge linking HUA and neuroinflammation. Chen et al. found that METTL3 was down-regulated in HUA mice, while METTL3 knockdown aggravated mouse hippocampal neuronal apoptosis and microglia overactivation through the NF-κB and NLRP3 inflammasome pathway, thus aggravating neuroinflammation (77).

#### 3.2.3 Diabetic complication

Type 2 diabetes mellitus results from the combination of two factors: defective insulin secretion by pancreatic β-cells and insulin resistance of peripheral target tissues (119). Diabetic kidney disease is the most common microvascular complication of diabetes. High glucose levels can elevate the expression of transforming growth factor β1, angiotensin II, cytokines, and advanced glycation end products, with adverse effects on a variety of renal cell types, including lipid oxidation, mitochondrial dysfunction, oxidation, endoplasmic reticulum stress, and fibrosis (120). Several epigenetic mechanisms, including DNA methylation, histone modification, and non-coding RNAs, have been implicated in the pathogenesis of diabetic kidney disease in recent years (120), and m6A modification is no exception. METTL3 is elevated in renal tubules from human biopsies and METTL3 silencing alleviated renal inflammation in tubular epithelial cells via the METTL3/TAB3 axis (78). METTL3 is also elevated in podocytes from renal biopsies from patients with diabetic nephropathy, while METTL3 knockout reduced the m6A levels of TIMP metallopeptidase inhibitor 2 (*TIMP2*) and downregulated its expression, which led to clear alleviation of inflammation and apoptosis via the Notch pathway in podocytes (79). Liu et al. found that m6A modification of phosphate and tension homology (*PTEN*) in podocytes was decreased under high glucose conditions (80). The underlying mechanism involved a high glucose-stimulated decrease in METTL3 expression in podocytes and decreased m6A modification of *PTEN*, which increased PTEN expression, and PTEN promoted podocyte pyroptosis through PI3K/Akt signaling. METTL3 contributes to the development of renal fibrosis by promoting inflammation. METTL3 facilitates *miR-21-5p* maturation, and *miR-21-5p* activates ERK/NF-κB signaling by suppressing sprouty RTK signaling antagonist 1 (SPRY1), which affects renal fibrosis progression (81).

### 3.3 Degenerative disease

#### 3.3.1 Osteoarthritis

OA is a chronic joint disease characterized by progressive cartilage degeneration, subchondral bone sclerosis, and synovial inflammation (121). Sang et al. found that METTL3 was decreased in patients with OA (83); however, Liu et al. showed that METTL3 was increased in experimental OA (82). METTL3 silencing reduced chondrocyte apoptosis, decreased inflammatory cytokine secretion and promoted extracellular matrix (ECM) degradation (82). METTL3 overexpression increased BCL2 expression through YTHDF1-mediated m6A modification and inhibited chondrocyte apoptosis and autophagy (84). Ribosomal protein L38 (RPL38) can positively regulate METTL3, while RPL38 knockdown increased levels of suppressor of cytokine signaling 2 (*SOCS2*) through decreasing its m6A level, mediated by METTL3, thereby reducing inflammation and apoptosis of chondrocytes and inhibiting ECM degradation (85). Chen et al. investigated the role of fibroblast-like synoviocyte (FLS) senescence in the development of OA and found that METTL3 knockdown increased the expression of autophagy-related 7 (ATG7), which inhibited GATA binding protein 4 (GATA4) to ameliorate FLS senescence and OA progression (86).

#### 3.3.2 Intervertebral disc degeneration

IDD is the main cause of back, neck, and radicular pain (122). The incidence rate of IDD rises with age, and its pathogenesis is related to senescence, apoptosis, inflammation levels, and ECM degradation of nucleus pulposus cells (NPCs) (123). Fang et al. found that METTL3 levels were increased in NPCs from patients with IDD (87), while METTL3 depletion resulted in the reduction of siah E3 ubiquitin protein ligase 1 (*SIAH1*) mRNA stability, which induced apoptosis, inflammation, and senescence, as well as decreasing ECM synthesis in NPCs (87).

### 3.4 Immune disease

#### 3.4.1 Rheumatoid arthritis

Rheumatoid arthritis (RA) is a chronic inflammatory autoimmune disease characterized by autoantibody production, synovial inflammation, cartilage damage, and bone erosion (124, 125). The etiology of rheumatoid arthritis is very complex. Current evidence shows that genotype, sex, environmental triggers, periodontal microbes, gastrointestinal microbes, and epigenetic modification are involved in the incidence and development of RA (125–127). Wang et al. found that METTL3 was significantly increased in peripheral blood mononuclear cells from patients with RA, and there were positive correlations between METTL3 levels and the markers of RA activity, C-reactive protein (CRP), and erythrocyte sedimentation rate (ESR) (88). Another *in vivo* experiment demonstrated that METTL3 was up-regulated in synovial tissue and FLSs from patients with RA and in an adjuvant-induced arthritis rat model (89). METTL3 silencing suppressed FLS proliferation, migration and invasion, and IL-6 expression, possibly by inhibiting the NF-κB pathway (89).

#### 3.4.2 Psoriasis

Psoriasis is a chronic, immune-mediated disorder, which can be provoked by non-specific triggers, such as mild trauma, sunburn, stress, infection (particularly streptococcal), alcohol, or drugs (128). Briefly, triggers stimulate keratinocytes to produce antimicrobial peptides, which in turn activate Th cells to polarize and release IFN-γ, IL-17A/IL-17F, and IL-22; these cytokines result in excessive proliferation and abnormal differentiation of keratinocytes, which release pro-inflammatory cytokines, further aggravating psoriasis (129–131). Recent evidence suggests that m6A epigenetic modification also contributes to the pathogenesis of psoriasis. Lower levels of m6A methylation and METTL3 were detected in psoriatic skin lesions than in healthy human skin samples (90), and levels of m6A methylation and METTL3 were negatively correlated with psoriasis severity (90). Further, compared with mice heterozygous for METTL3 knockout, wild-type control mice showed ameliorated psoriasis-like clinical and pathological manifestations (90). Moreover, KEGG analysis suggests that METTL3 may play a role in the pathogenesis of psoriasis through the WNT pathway (90).

#### 3.4.3 Inflammatory bowel disease

IBD describes chronic inflammatory disorders of the intestine (132). Maialen et al. used an online genome-wide association study and m6A transcription databases to screen five IBD-related single nucleotide polymorphisms in the *UBE2L3*, *SLC22A4*, *TCF19*, *C6orf47*, and *SNAPC4* genes, suggesting that m6A methylation may contribute to IBD pathogenesis (133). Subsequently, METTL3 was found to be elevated in ulcerative colitis tissue from patients with IBD, a dextran sulfate sodium-induced IBD mouse model, and LPS-treated MODE-K cells, while *in vivo* and *in vitro* knockdown of METTL3 reduced levels of proinflammatory cytokines and inflammatory enzymes and inhibited p65 phosphorylation (91).

### 3.5 Infectious diseases

#### 3.5.1 Sepsis

Sepsis is a life-threatening organ dysfunction resulting from dysregulated host responses to infection (134); it can be caused by almost any infectious organism, and the incidence rates of sepsis due to infection by gram-negative and gram-positive bacteria are almost equal (135). The most common infection sites leading to sepsis are the lung, abdomen, blood, kidney, and genitourinary tract (136). According to traditional understanding, sepsis is an overwhelming systemic inflammatory response to infection, accompanied by immunosuppression and secondary infection (137). Sepsis-induced organ dysfunction includes cardiomyopathy, encephalopathy, and cognitive impairment, as well as liver injury, acute kidney injury, and acute respiratory distress syndrome (ARDS). Although many studies have shown that sepsis can cause organ dysfunction by interfering with microcirculation, ischemia, hypoxia, mitophagy, and cell apoptosis, the exact mechanism involved is not fully understood (138).

Aortic injury during sepsis leads to insufficient organ perfusion, but the specific mechanism has not been fully elucidated. Shen et al. found that levels of m6A and METTL3 expression were significantly decreased in aortic tissue from an LPS-induced sepsis rat model (92). According to high-throughput sequencing, KEGG, and GO enrichment analyses, genes affected by m6A modification were related to cation channels, particularly TRP channels, indicating that METTL3 may aggravate vascular endothelial cell inflammation during sepsis by interfering with calcium ion transport (92).

NETs were increased in humans and mice with sepsis-associated ARDS and positively associated with disease severity level, indicating that NETs are harmful in sepsis-associated ARDS (93). Further, Zhang et al. found that NETs upregulated METTL3 expression, leading to methylation of glutathione peroxidase 4 (*GPX4*) mRNA and decreased GPX4 expression via accelerated degradation of *GPX4* mRNA mediated by YTHDF2, with consequent ferroptosis of alveolar epithelial cells (93). Qu et al. showed that NETs could promote METTL3-mediated m6A methylation of sirtuin 1 (*SIRT1*) mRNA in alveolar epithelial cells, leading to abnormal cell autophagy and deterioration of ARDS (94). Chen et al. found that METTL3 expression was reduced in LPS-induced ARDS mice and LPS-stimulated human pulmonary vascular endothelial cells (95). METTL3 downregulation destroyed endothelial permeability and led to higher inflammatory cytokine expression by reducing the stability of *Trim59* mRNA and activating the NF-κB pathway. *CircN4bp1* up-regulated the expression of the enhancer of the zeste 2 polycomb repressive complex 2 subunit (EZH2) by combining with *miR-138-5p*, thus promoting M1 macrophage polarization and aggravating the degree of lung injury in ARDS mice (96). Further, Zhao et al. found that the m6A levels in *circN4bp1* were increased in ARDS mice and that the inflammatory response could be blocked by inhibiting METTL3, suggesting that METTL3 might be involved in regulating *circN4bp1* expression (96).

#### 3.5.2 Pneumonia

Pneumonia is a common acute respiratory infection caused by many types of microorganisms, including bacteria, respiratory viruses, and fungi, and primarily occurs in children < 5 years old and elderly adults with underlying chronic conditions (139). Yang et al. found that METTL3 was increased in pediatric patients with pneumonia and WI-38 cells, while METTL3 knockdown inhibited the inflammatory response and apoptosis by reducing the expression of EZH2 and the JAK/STAT3 pathway (97). In another study, METTL3 knockdown reduced the translation of *STAT2* mRNA mediated by YTHDF1 and alleviated LPS-induced inflammatory damage (98).

#### 3.5.3 Other infectious diseases

When a virus invades, innate immune and epithelial cells can recognize viral structures, such as nucleic acids, peptides, and lipoproteins, leading to primary activation of interferon regulatory factor (IRF) 3/7 and the NF-κB signaling pathway, which induces the production of pro-inflammatory cytokines and interferon (140). Interferon induces interferon-stimulated gene (ISG) transcription by activating the JAK1/STAT1/STAT2 pathway (140). Some viruses can modify host cell genes through epigenetic strategies, to enhance replication and survival. Epstein–Barr virus infection can alter the m6A-modified epitranscriptome of host B cells and upregulate mRNA and protein levels of fas cell surface death receptor (FAS) but downregulate toll-like receptor 9 (TLR9), which are related to apoptosis and virus infection (141). Wang et al. found that METTL3-deficient mice had stronger resistance to rotavirus. METTL3 knockout in intestinal epithelial cells reduced m6A modification of *IRF7* and increased its expression, leading to the upregulation of interferons and ISGs (109). Li et al. found that METTL3 depletion of host cells decreased m6A levels of SARS-CoV-2 and the host genome, subsequently increasing innate immune signaling and inflammatory gene expression (99).

Fungal keratitis (FK) is an aggressive infectious corneal disease caused by pathogenic fungi (142). METTL3 was increased in corneas of an FK mouse model and in fungi-infected corneal stromal cells, while METTL3 knockdown attenuated the FK inflammatory response by inhibiting the PI3K/AKT or TRAF6/NF-κB pathways (100, 101).

In addition, some studies of inflammation have been conducted using LPS-stimulated cells. METTL3 protein expression was elevated in LPS-induced retinal pigment epithelium cells, and silencing METTL3 increased the expression of nuclear receptor subfamily 2 group F member 1 (NR2F1) and promoted IL-6 secretion (102). Further, METTL3 was up-regulated in LPS-stimulated human dental pulp cells, and its knockdown reduced inflammatory cytokine secretion through the NF-κB and MAPK pathways (103).

### 3.6 Tumors

Chronic inflammation is a risk factor for tumor development, as it provides a microenvironment conducive to tumorigenesis, tumor development, and metastasis (143). A deep understanding of the regulatory mechanisms in the inflammatory tumor microenvironment will help to develop more effective immunotherapy. Most tumors recruit tumor-associated macrophages (TAMs) to create a microenvironment favorable for tumor growth. METTL3 has recently been shown to affect the recruitment and polarization of TAMs, with METTL3-deficient mice exhibiting increased infiltration of M1/M2-like TAMs and regulatory T cells into tumors compared with wild-type mice (104). METTL3 knockout promoted M1 and M2 polarization of macrophages by activating the NF-κB/STAT3 pathways (104). Co-culture of monocyte and glioma cells demonstrated that glioma cells overexpressing METTL3 inhibited SOCS2 expression and M1 polarization of monocytes (110), while SOCS2 overexpression could rescue this inhibition. Knockdown of METTL3 in TAMs infiltrating prostate cancer promotes M2 macrophage polarization by activating STAT6 (106). Further, M2-TAMs can promote lung adenocarcinoma immuno-resistance by enhancing METTL3-mediated m6A methylation (107), as well as inducing oxaliplatin resistance by increasing METTL3 in colorectal cancer cells (108).

In addition, METTL3 regulates the infiltration of tumor-associated neutrophils. Silencing METTL3 caused papillary thyroid cancer cells to secrete more pro-inflammatory factors, particularly IL-8, which can recruit infiltration of tumor-associated neutrophils. The NF-κB pathway inhibitor, BAY 11-7082, inhibits IL-8 production by tumor cells and neutrophil infiltration, as well as inhibiting tumor growth (105).

## 4 Therapeutic application of METTL3

The study of METTL3 expression changes and related pathogenic processes in inflammatory diseases provides clues for the development of effective drugs and informs novel diagnostic strategies (**Table 3**).

**Table 3:** Effects of natural products, synthetic compounds, and biological agents on METTL3.

Category	Examples	Effect	*In vivo*	*In vitro*	Ref
Natural Products	Emodin	Upregulation of METTL3	–	LPS-treated 1321N1 cells	(144)
	Cinnamaldehyde	Upregulation of METTL3	–	Free fatty acids induced AML12	(145)
	Total flavones of Abelmoschus manihot	Upregulation of METTL3	–	High glucose treated MPC-5 cells	(80)
	Resveratrol	Downregulation of METTL3	Mice fed with high-fat diet	–	(146)
	Resveratrol	Upregulation of METTL3	Mice fed with high-fat diet	–	(147)
Synthetic compounds	Cpd-564	Downregulation of METTL3	Cisplatin- and I/R-induced AKI mouse model	Cisplatin-treated HK2 cells	(78)
	S-adenosylhomocysteine (SAH)	Downregulation of METTL3	Monosodium iodoacetate induced TMJ OA mice	TNF-α induced chondrocytes	(84)
	Methylation inhibitor cycloleucine	No effects	Collagenase-induced OA mice	–	(82)
	Methyl donor betaine	No effects	Collagenase-induced OA mice	–	(82)
Biological agents	miR-1208 within hucMSCs-EVs	Downregulation of METTL3	DMM-induced OA knee mice model	LPS and nigericin-treated THP-1	(148)

The therapeutic potential of METTL3 is mainly as a pharmacological target for natural plant medicinal ingredients. Emodin, the main active component of rhubarb, exhibits significant anti-inflammatory activity (149) and can promote METTL3 expression, which downregulates NLRP3 by increasing its m6A levels in LPS-treated 1321N1 cells, thus suppressing pyroptosis and proinflammatory cytokine secretion (144). Cinnamaldehyde (CA) is a natural ingredient extracted from the herb, *Ramulus cinnamomi*, which has beneficial functions in lipid metabolism and inflammation of the liver (150). Xu et al. found that METTL3 was decreased in mouse hepatocytes stimulated by free fatty acids and aggravated cell steatosis by down-regulating CYP4F40, which could be compensated by CA (145), where CA can promote METTL3 expression and improve steatosis (145). Total flavones isolated from *Abelmoschus manihot* (TFA) have been used to treat kidney disease (151). Liu et al. found that TFA could upregulate METTL3 expression in podocytes stimulated by high glucose and that METTL3 overexpression promoted PTEN expression via m6A modification of *PTEN*, thereby inhibiting podocyte pyroptosis mediated by PI3K/Akt signaling (80). Resveratrol is a natural phytoalexin, present in grapes, peanuts, and their derivatives (152), which has a neuroprotective role (153). Izquierdo et al. found that adding resveratrol to the high-fat diet of mice could clearly up-regulate m6A levels in the hippocampus of their offspring and reduce expression of METTL3 and FTO, although the significance and impact of METTL3 reduction have not been studied (146). In another study, resveratrol intake in mice fed a high-fat diet could increase METTL3 in the liver tissue, reduce liver fat accumulation, and increase *PPARα* mRNA levels (147); however, the molecular mechanism underlying METTL3 activity in resveratrol-regulated liver lipid homeostasis remains to be determined.

The efficacy of METTL3 inhibitors has also been investigated. Cpd-564, a new METTL3 inhibitor identified by Wang et al., exerted reno-protective effects in cisplatin-treated HK2 cells and an acute kidney injury (AKI) mouse model (78). Cpd-564 inhibited TAB3 expression and inflammatory responses in HK2 cells tubule damage was alleviated in an AKI model after Cpd-564 administration; however, METTL3 inhibition may be harmful to temporomandibular joint (TMJ) OA. The METTL3 inhibitor, S-adenosylhomocysteine, promotes chondrocyte apoptosis and autophagy induced by TNF-α, and aggravated chondrocyte degeneration in TMJ OA mice (84). In addition, the methylation inhibitor, cycloleucine, can reduce IL-8 and IL-6 and promote collagen type II levels in OA cartilage tissue (82).

Moreover, METTL3 may be a target in OA treatment using extracellular vesicles derived from human umbilical cord mesenchymal stem cells (hucMSCs-EVs); *miR-1208* in hucMSCs-EVs reduced m6A levels in *NLRP3* mRNA by down-regulating METTL3 expression in macrophages, which prevented NLRP3 inflammasome activation and exerted anti-inflammatory effects (148).

In addition, METTL3 may serve as a novel biomarker for RA diagnosis. Wang et al. found that increased METTL3 in RA patients was positively correlated with CRP and ESR (88); however, its sensitivity and specificity require further verification.

## 5 Conclusion

The study of METTL3-mediated m6A modification brings new insights into the pathogenesis of inflammatory diseases. METTL3 is upregulated in most inflammatory diseases, indicating that it has pro-inflammatory effects; however, opposite expression trends in METTL3 expression have also been observed in the same diseases, including sepsis-related ARDS and OA, indicating that the effects of its expression may be related to cell type, modeling methods, disease status, or other potential regulatory factors. Moreover, METTL3 can regulate different target genes in the same disease, which challenges the accuracy and safety of drug development. In addition, METTL3 can regulate macrophage M1 polarization, implying that METTL3 is a potential therapeutic target in macrophage-related diseases. As METTL3 can regulate the infiltration and polarization of TAMs and tumor-associated neutrophils, it is meaningful to study the therapeutic application of METTL3 in anti-tumor immune responses. There have been promising studies of natural drugs targeting METTL3 in the treatment of inflammatory diseases, although their detailed pharmacological effects are not yet fully understood. Whether agents targeting METTL3 are natural plant-derived ingredients, chemically synthesized drugs, or biological agents, their therapeutic prospects warrant further research.

## Author contributions

BS conceived and drafted the manuscript. BS and YZ designed and drew the figures. YC, JZ, and CX assisted in searching and sorting articles. YP and XZ reviewed and provided suggestions for the manuscript. JL reviewed and revised the manuscript. All authors contributed to the article and approved the submitted version.

## References

[1] Medzhitov R. Inflammation 2010: new adventures of an old flame. Cell (2010) 140:771–6. doi: 10.1016/j.cell.2010.03.006 20303867

[2] Medzhitov R. Origin and physiological roles of inflammation. Nature (2008) 454:428–35. doi: 10.1038/nature07201 18650913

[3] Kotas ME Medzhitov R. Homeostasis, inflammation, and disease susceptibility. Cell (2015) 160:816–27. doi: 10.1016/j.cell.2015.02.010 PMC4369762 25723161

[4] Weiss U. Inflammation. Nature (2008) 454:427–7. doi: 10.1038/454427a 18650912

[5] Takeuchi O Akira S. Pattern recognition receptors and inflammation. Cell (2010) 140:805–20. doi: 10.1016/j.cell.2010.01.022 20303872

[6] Netea MG Balkwill F Chonchol M Cominelli F Donath MY Giamarellos-Bourboulis EJ. A guiding map for inflammation. Nat Immunol (2017) 18:826–31. doi: 10.1038/ni.3790 PMC5939996 28722720

[7] Headland SE Norling LV. The resolution of inflammation: Principles and challenges. Semin Immunol (2015) 27:149–60. doi: 10.1016/j.smim.2015.03.014 25911383

[8] Furman D Campisi J Verdin E Carrera-Bastos P Targ S Franceschi C. Chronic inflammation in the etiology of disease across the life span. Nat Med (2019) 25:1822–32. doi: 10.1038/s41591-019-0675-0 PMC7147972 31806905

[9] Liston A Masters SL. Homeostasis-altering molecular processes as mechanisms of inflammasome activation. Nat Rev Immunol (2017) 17:208–14. doi: 10.1038/nri.2016.151 28163301

[10] Sugimoto MA Sousa LP Pinho V Perretti M Teixeira MM. Resolution of inflammation: what controls its onset? Front Immunol (2016) 7:160. doi: 10.3389/fimmu.2016.00160 27199985 PMC4845539

[11] Luo J Xu T Sun K. N6-methyladenosine RNA modification in inflammation: roles, mechanisms, and applications. Front Cell Dev Biol (2021) 9:670711. doi: 10.3389/fcell.2021.670711 34150765 PMC8213350

[12] Tuck MT. The formation of internal 6-methyladenine residues in eucaryotic messenger RNA. Int J Biochem (1992) 24:379–86. doi: 10.1016/0020-711x(92)90028-y 1551452

[13] Wei CM Gershowitz A Moss B. 5’-Terminal and internal methylated nucleotide sequences in HeLa cell mRNA. Biochemistry (1976) 15:397–401. doi: 10.1021/bi00647a024 174715

[14] Dominissini D Moshitch-Moshkovitz S Schwartz S Salmon-Divon M Ungar L Osenberg S. Topology of the human and mouse m6A RNA methylomes revealed by m6A-seq. Nature (2012) 485:201–6. doi: 10.1038/nature11112 22575960

[15] Meyer KD Saletore Y Zumbo P Elemento O Mason CE Jaffrey SR. Comprehensive analysis of mRNA methylation reveals enrichment in 3’ UTRs and near stop codons. Cell (2012) 149:1635–46. doi: 10.1016/j.cell.2012.05.003 PMC3383396 22608085

[16] Zaccara S Ries RJ Jaffrey SR. Reading, writing and erasing mRNA methylation. Nat Rev Mol Cell Biol (2019) 20:608–24. doi: 10.1038/s41580-019-0168-5 31520073

[17] Bokar JA Shambaugh ME Polayes D Matera AG Rottman FM. Purification and cDNA cloning of the AdoMet-binding subunit of the human mRNA (N6-adenosine)-methyltransferase. RNA (1997) 3:1233–47. PMC1369564 9409616

[18] Liu J Yue Y Han D Wang X Fu Y Zhang L. A METTL3-METTL14 complex mediates mamMalian nuclear RNA N6-adenosine methylation. Nat Chem Biol (2014) 10:93–5. doi: 10.1038/nchembio.1432 PMC3911877 24316715

[19] Pendleton KE Chen B Liu K Hunter OV Xie Y Tu BP. The U6 snRNA m6A Methyltransferase METTL16 Regulates SAM Synthetase Intron Retention. Cell (2017) 169:824–835.e14. doi: 10.1016/j.cell.2017.05.003 28525753 PMC5502809

[20] Ping X-L Sun B-F Wang L Xiao W Yang X Wang W-J. MamMalian WTAP is a regulatory subunit of the RNA N6-methyladenosine methyltransferase. Cell Res (2014) 24:177–89. doi: 10.1038/cr.2014.3 PMC3915904 24407421

[21] Schwartz S Mumbach MR Jovanovic M Wang T Maciag K Bushkin GG. Perturbation of m6A writers reveals two distinct classes of mRNA methylation at internal and 5’ sites. Cell Rep (2014) 8:284–96. doi: 10.1016/j.celrep.2014.05.048 PMC4142486 24981863

[22] Patil DP Chen C-K Pickering BF Chow A Jackson C Guttman M. m(6)A RNA methylation promotes XIST-mediated transcriptional repression. Nature (2016) 537:369–73. doi: 10.1038/nature19342 PMC5509218 27602518

[23] Wen J Lv R Ma H Shen H He C Wang J. Zc3h13 regulates nuclear RNA m6A methylation and mouse embryonic stem cell self-renewal. Mol Cell (2018) 69:1028–1038.e6. doi: 10.1016/j.molcel.2018.02.015 29547716 PMC5858226

[24] Jia G Fu Y Zhao X Dai Q Zheng G Yang Y. N6-methyladenosine in nuclear RNA is a major substrate of the obesity-associated FTO. Nat Chem Biol (2011) 7:885–7. doi: 10.1038/nchembio.687 PMC3218240 22002720

[25] Zheng G Dahl JA Niu Y Fedorcsak P Huang C-M Li CJ. ALKBH5 is a mamMalian RNA demethylase that impacts RNA metabolism and mouse fertility. Mol Cell (2013) 49:18–29. doi: 10.1016/j.molcel.2012.10.015 23177736 PMC3646334

[26] Xiao W Adhikari S Dahal U Chen Y-S Hao Y-J Sun B-F. Nuclear m(6)A Reader YTHDC1 Regulates mRNA Splicing. Mol Cell (2016) 61:507–19. doi: 10.1016/j.molcel.2016.01.012 26876937

[27] Hsu PJ Zhu Y Ma H Guo Y Shi X Liu Y. Ythdc2 is an N6-methyladenosine binding protein that regulates mamMalian spermatogenesis. Cell Res (2017) 27:1115–27. doi: 10.1038/cr.2017.99 PMC5587856 28809393

[28] Wang X Lu Z Gomez A Hon GC Yue Y Han D. N6-methyladenosine-dependent regulation of messenger RNA stability. Nature (2014) 505:117–20. doi: 10.1038/nature12730 PMC3877715 24284625

[29] Wang X Zhao BS Roundtree IA Lu Z Han D Ma H. N(6)-methyladenosine modulates messenger RNA translation efficiency. Cell (2015) 161:1388–99. doi: 10.1016/j.cell.2015.05.014 PMC4825696 26046440

[30] YTHDF3 facilitates translation and decay of N6-methyladenosine-modified RNA - PubMed. (Accessed February 10, 2023). 10.1038/cr.2017.15 PMC5339834 28106072

[31] Wu B Su S Patil DP Liu H Gan J Jaffrey SR. Molecular basis for the specific and multivariant recognitions of RNA substrates by human hnRNP A2/B1. Nat Commun (2018) 9:420. doi: 10.1038/s41467-017-02770-z 29379020 PMC5789076

[32] Liu N Dai Q Zheng G He C Parisien M Pan T. N(6)-methyladenosine-dependent RNA structural switches regulate RNA-protein interactions. Nature (2015) 518:560–4. doi: 10.1038/nature14234 PMC4355918 25719671

[33] Huang H Weng H Sun W Qin X Shi H Wu H. Recognition of RNA N6-methyladenosine by IGF2BP proteins enhances mRNA stability and translation. Nat Cell Biol (2018) 20:285–95. doi: 10.1038/s41556-018-0045-z PMC5826585 29476152

[34] Roundtree IA Evans ME Pan T He C. Dynamic RNA modifications in gene expression regulation. Cell (2017) 169:1187–200. doi: 10.1016/j.cell.2017.05.045 PMC5657247 28622506

[35] Wang X Feng J Xue Y Guan Z Zhang D Liu Z. Structural basis of N(6)-adenosine methylation by the METTL3-METTL14 complex. Nature (2016) 534:575–8. doi: 10.1038/nature18298 27281194

[36] Schöller E Weichmann F Treiber T Ringle S Treiber N Flatley A. Interactions, localization, and phosphorylation of the m6A generating METTL3-METTL14-WTAP complex. RNA N Y N (2018) 24:499–512. doi: 10.1261/rna.064063.117 PMC5855951 29348140

[37] Liu S Zhuo L Wang J Zhang Q Li Q Li G. METTL3 plays multiple functions in biological processes. Am J Cancer Res (2020) 10:1631–46. PMC7339281 32642280

[38] Epelman S Lavine KJ Randolph GJ. Origin and functions of tissue macrophages. Immunity (2014) 41:21–35. doi: 10.1016/j.immuni.2014.06.013 25035951 PMC4470379

[39] Cassetta L Cassol E Poli G. Macrophage polarization in health and disease. Sci World J (2011) 11:2391–402. doi: 10.1100/2011/213962 PMC3236674 22194670

[40] Bashir S Sharma Y Elahi A Khan F. Macrophage polarization: the link between inflammation and related diseases. Inflammation Res (2016) 65:1–11. doi: 10.1007/s00011-015-0874-1 26467935

[41] Jetten N Verbruggen S Gijbels MJ Post MJ De Winther MPJ Donners MMPC. Anti-inflammatory M2, but not pro-inflammatory M1 macrophages promote angiogenesis in *vivo* . Angiogenesis (2014) 17:109–18. doi: 10.1007/s10456-013-9381-6 24013945

[42] Locati M Curtale G Mantovani A. Diversity, mechanisms, and significance of macrophage plasticity. Annu Rev Pathol (2020) 15:123–47. doi: 10.1146/annurev-pathmechdis-012418-012718 PMC7176483 31530089

[43] Shapouri-Moghaddam A Mohammadian S Vazini H Taghadosi M Esmaeili S-A Mardani F. Macrophage plasticity, polarization, and function in health and disease. J Cell Physiol (2018) 233:6425–40. doi: 10.1002/jcp.26429 PMC13482560 29319160

[44] Li Z Xu Q Huangfu N Chen X Zhu J. Mettl3 promotes oxLDL-mediated inflammation through activating STAT1 signaling. J Clin Lab Anal (2022) 36:e24019. doi: 10.1002/jcla.24019 34825733 PMC8761454

[45] Sun Z Chen W Wang Z Wang S Zan J Zheng L. Matr3 reshapes m6A modification complex to alleviate macrophage inflammation during atherosclerosis. Clin Immunol Orlando Fla (2022) 245:109176. doi: 10.1016/j.clim.2022.109176 36368640

[46] Zhang X Li X Jia H An G Ni J. The m(6)A methyltransferase METTL3 modifies PGC-1α mRNA promoting mitochondrial dysfunction and oxLDL-induced inflammation in monocytes. J Biol Chem (2021) 297:101058. doi: 10.1016/j.jbc.2021.101058 34375639 PMC8406003

[47] Guo M Yan R Ji Q Yao H Sun M Duan L. IFN regulatory Factor-1 induced macrophage pyroptosis by modulating m6A modification of circ_0029589 in patients with acute coronary syndrome. Int Immunopharmacol (2020) 86:106800. doi: 10.1016/j.intimp.2020.106800 32674051

[48] Cai Y Yu R Kong Y Feng Z Xu Q. METTL3 regulates LPS-induced inflammatory response via the NOD1 signaling pathway. Cell Signal (2022) 93:110283. doi: 10.1016/j.cellsig.2022.110283 35176453

[49] Tong J Wang X Liu Y Ren X Wang A Chen Z. Pooled CRISPR screening identifies m6A as a positive regulator of macrophage activation. Sci Adv (2021) 7:eabd4742. doi: 10.1126/sciadv.abd4742 33910903 PMC8081357

[50] Nayak D Roth TL McGavern DB. Microglia development and function. Annu Rev Immunol (2014) 32:367–402. doi: 10.1146/annurev-immunol-032713-120240 24471431 PMC5001846

[51] Wen L Sun W Xia D Wang Y Li J Yang S. The m6A methyltransferase METTL3 promotes LPS-induced microglia inflammation through TRAF6/NF-κB pathway. Neuroreport (2022) 33:243–51. doi: 10.1097/WNR.0000000000001550 33165191

[52] Liu Y Liu Z Tang H Shen Y Gong Z Xie N. The N6-methyladenosine (m6A)-forming enzyme METTL3 facilitates M1 macrophage polarization through the methylation of STAT1 mRNA. Am J Physiol Cell Physiol (2019) 317:C762–75. doi: 10.1152/ajpcell.00212.2019 31365297

[53] Zheng L Chen X Yin Q Gu J Chen J Chen M. RNA-m6A modification of HDGF mediated by Mettl3 aggravates the progression of atherosclerosis by regulating macrophages polarization via energy metabolism reprogramming. Biochem Biophys Res Commun (2022) 635:120–7. doi: 10.1016/j.bbrc.2022.10.032 36265285

[54] Groh L Keating ST Joosten LAB Netea MG Riksen NP. Monocyte and macrophage immunometabolism in atherosclerosis. Semin Immunopathol (2018) 40:203–14. doi: 10.1007/s00281-017-0656-7 PMC5809534 28971272

[55] Liew PX Kubes P. The neutrophil’s role during health and disease. Physiol Rev (2019) 99:1223–48. doi: 10.1152/physrev.00012.2018 30758246

[56] Luo S Liao C Zhang L Ling C Zhang X Xie P. METTL3-mediated m6A mRNA methylation regulates neutrophil activation through targeting TLR4 signaling. Cell Rep (2023) 42:112259. doi: 10.1016/j.celrep.2023.112259 36920907

[57] Liu J Zhang X Cheng Y Cao X. Dendritic cell migration in inflammation and immunity. Cell Mol Immunol (2021) 18:2461. doi: 10.1038/s41423-021-00726-4 34302064 PMC8298985

[58] Wang H Hu X Huang M Liu J Gu Y Ma L. Mettl3-mediated mRNA m6A methylation promotes dendritic cell activation. Nat Commun (2019) 10:1898. doi: 10.1038/s41467-019-09903-6 31015515 PMC6478715

[59] Wu H Xu Z Wang Z Ren Z Li L Ruan Y. Dendritic cells with METTL3 gene knockdown exhibit immature properties and prolong allograft survival. Genes Immun (2020) 21:193–202. doi: 10.1038/s41435-020-0099-3 32457372

[60] Harrington LE Hatton RD Mangan PR Turner H Murphy TL Murphy KM. Interleukin 17-producing CD4+ effector T cells develop via a lineage distinct from the T helper type 1 and 2 lineages. Nat Immunol (2005) 6:1123–32. doi: 10.1038/ni1254 16200070

[61] Yasuda K Takeuchi Y Hirota K. The pathogenicity of Th17 cells in autoimmune diseases. Semin Immunopathol (2019) 41:283–97. doi: 10.1007/s00281-019-00733-8 30891627

[62] Park H Li Z Yang XO Chang SH Nurieva R Wang Y-H. A distinct lineage of CD4 T cells regulates tissue inflammation by producing interleukin 17. Nat Immunol (2005) 6:1133–41. doi: 10.1038/ni1261 PMC1618871 16200068

[63] Zhao L Liu Y Ma B Liu X Wei R Nian H. METTL3 inhibits autoreactive Th17 cell responses in experimental autoimmune uveitis via stabilizing ASH1L mRNA. FASEB J Off Publ Fed Am Soc Exp Biol (2023) 37:e22803. doi: 10.1096/fj.202201548R 36753389

[64] Cai Y Chen X Huang C Chen Y Zhang C Huang Z. Alteration of m6A-tagged RNA profiles in bone originated from periprosthetic joint infection. J Clin Med (2023) 12:2863. doi: 10.3390/jcm12082863 37109200 PMC10146075

[65] Björkström NK Strunz B Ljunggren H-G. Natural killer cells in antiviral immunity. Nat Rev Immunol (2022) 22:112–23. doi: 10.1038/s41577-021-00558-3 PMC8194386 34117484

[66] Vivier E Tomasello E Baratin M Walzer T Ugolini S. Functions of natural killer cells. Nat Immunol (2008) 9:503–10. doi: 10.1038/ni1582 18425107

[67] Screpanti V Wallin RPA Grandien A Ljunggren H-G. Impact of FASL-induced apoptosis in the elimination of tumor cells by NK cells. Mol Immunol (2005) 42:495–9. doi: 10.1016/j.molimm.2004.07.033 15607805

[68] Song H Song J Cheng M Zheng M Wang T Tian S. METTL3-mediated m(6)A RNA methylation promotes the anti-tumour immunity of natural killer cells. Nat Commun (2021) 12:5522. doi: 10.1038/s41467-021-25803-0 34535671 PMC8448775

[69] Dong G Yu J Shan G Su L Yu N Yang S. N6-Methyladenosine Methyltransferase METTL3 Promotes Angiogenesis and Atherosclerosis by Upregulating the JAK2/STAT3 Pathway via m6A Reader IGF2BP1. Front Cell Dev Biol (2021) 9:731810. doi: 10.3389/fcell.2021.731810 34950654 PMC8689138

[70] Li B Zhang T Liu M Cui Z Zhang Y Liu M. RNA N(6)-methyladenosine modulates endothelial atherogenic responses to disturbed flow in mice. eLife (2022) 11:e69906. doi: 10.7554/eLife.69906 35001873 PMC8794471

[71] Chien C-S Li JY-S Chien Y Wang M-L Yarmishyn AA Tsai P-H. METTL3-dependent N6-methyladenosine RNA modification mediates the atherogenic inflammatory cascades in vascular endothelium. Proc Natl Acad Sci U.S.A. (2021) 118:e2025070118. doi: 10.1073/pnas.2025070118 33579825 PMC7896299

[72] Qi L Hu H Wang Y Hu H Wang K Li P. New insights into the central sympathetic hyperactivity post-myocardial infarction: Roles of METTL3-mediated m(6) A methylation. J Cell Mol Med (2022) 26:1264–80. doi: 10.1111/jcmm.17183 PMC8831944 35040253

[73] Qi L Wang Y Hu H Li P Hu H Li Y. m(6)A methyltransferase METTL3 participated in sympathetic neural remodeling post-MI via the TRAF6/NF-κB pathway and ROS production. J Mol Cell Cardiol (2022) 170:87–99. doi: 10.1016/j.yjmcc.2022.06.004 35717715

[74] Qin Y Li B Arumugam S Lu Q Mankash SM Li J. m6A mRNA methylation-directed myeloid cell activation controls progression of NAFLD and obesity. Cell Rep (2021) 37:109968. doi: 10.1016/j.celrep.2021.109968 34758326 PMC8667589

[75] Li X Yuan B Lu M Wang Y Ding N Liu C. The methyltransferase METTL3 negatively regulates nonalcoholic steatohepatitis (NASH) progression. Nat Commun (2021) 12:7213. doi: 10.1038/s41467-021-27539-3 34893641 PMC8664922

[76] Shu B Zhou Y-X Li H Zhang R-Z He C Yang X. The METTL3/MALAT1/PTBP1/USP8/TAK1 axis promotes pyroptosis and M1 polarization of macrophages and contributes to liver fibrosis. Cell Death Discovery (2021) 7:368. doi: 10.1038/s41420-021-00756-x 34839365 PMC8627510

[77] Chen Y Cao P Xiao Z Ruan Z. m(6)A methyltransferase METTL3 relieves cognitive impairment of hyPeruricemia mice via inactivating MyD88/NF-κB pathway mediated NLRP3-ASC-Caspase1 inflammasome. Int Immunopharmacol (2022) 113:109375. doi: 10.1016/j.intimp.2022.109375 36461592

[78] Wang J-N Wang F Ke J Li Z Xu C-H Yang Q. Inhibition of METTL3 attenuates renal injury and inflammation by alleviating TAB3 m6A modifications via IGF2BP2-dependent mechanisms. Sci Transl Med (2022) 14:eabk2709. doi: 10.1126/scitranslmed.abk2709 35417191

[79] Jiang L Liu X Hu X Gao L Zeng H Wang X. METTL3-mediated m(6)A modification of TIMP2 mRNA promotes podocyte injury in diabetic nephropathy. Mol Ther J Am Soc Gene Ther (2022) 30:1721–40. doi: 10.1016/j.ymthe.2022.01.002 PMC9077313 34995800

[80] Liu B-H Tu Y Ni G-X Yan J Yue L Li Z-L. Total Flavones of Abelmoschus manihot Ameliorates Podocyte Pyroptosis and Injury in High Glucose Conditions by Targeting METTL3-Dependent m(6)A Modification-Mediated NLRP3-Inflammasome Activation and PTEN/PI3K/Akt Signaling. Front Pharmacol (2021) 12:667644. doi: 10.3389/fphar.2021.667644 34335245 PMC8319635

[81] Liu E Lv L Zhan Y Ma Y Feng J He Y. METTL3/N6-methyladenosine/ miR-21-5p promotes obstructive renal fibrosis by regulating inflammation through SPRY1/ERK/NF-κB pathway activation. J Cell Mol Med (2021) 25:7660–74. doi: 10.1111/jcmm.16603 PMC8358893 34164910

[82] Liu Q Li M Jiang L Jiang R Fu B. METTL3 promotes experimental osteoarthritis development by regulating inflammatory response and apoptosis in chondrocyte. Biochem Biophys Res Commun (2019) 516:22–7. doi: 10.1016/j.bbrc.2019.05.168 31186141

[83] Sang W Xue S Jiang Y Lu H Zhu L Wang C. METTL3 involves the progression of osteoarthritis probably by affecting ECM degradation and regulating the inflammatory response. Life Sci (2021) 278:119528. doi: 10.1016/j.lfs.2021.119528 33894271

[84] He Y Wang W Xu X Yang B Yu X Wu Y. Mettl3 inhibits the apoptosis and autophagy of chondrocytes in inflammation through mediating Bcl2 stability via Ythdf1-mediated m(6)A modification. Bone (2022) 154:116182. doi: 10.1016/j.bone.2021.116182 34530171

[85] Shi L Hu H Sun P Li Z Ji L Liu S. RPL38 knockdown inhibits the inflammation and apoptosis in chondrocytes through regulating METTL3-mediated SOCS2 m6A modification in osteoarthritis. Inflammation Res Off J Eur Histamine Res Soc Al (2022) 71:977–89. doi: 10.1007/s00011-022-01579-x 35596790

[86] Chen X Gong W Shao X Shi T Zhang L Dong J. METTL3-mediated m(6)A modification of ATG7 regulates autophagy-GATA4 axis to promote cellular senescence and osteoarthritis progression. Ann Rheum Dis (2022) 81:87–99. doi: 10.1136/annrheumdis-2021-221091 34706873

[87] Fang S Zeng F Chen R Li M. SIAH1 promotes senescence and apoptosis of nucleus pulposus cells to exacerbate disc degeneration through ubiquitinating XIAP. Tissue Cell (2022) 76:101820. doi: 10.1016/j.tice.2022.101820 35580525

[88] Wang J Yan S Lu H Wang S Xu D. METTL3 attenuates LPS-induced inflammatory response in macrophages via NF-κB signaling pathway. Mediators Inflammation (2019) 2019:3120391. doi: 10.1155/2019/3120391 PMC6854952 31772500

[89] Shi W Zheng Y Luo S Li X Zhang Y Meng X. METTL3 promotes activation and inflammation of FLSs through the NF-κB signaling pathway in rheumatoid arthritis. Front Med (2021) 8:607585. doi: 10.3389/fmed.2021.607585 PMC8290917 34295905

[90] Wang Y Huang J Jin H. Reduction of methyltransferase-like 3-mediated RNA N6-methyladenosine exacerbates the development of psoriasis vulgaris in imiquimod-induced psoriasis-like mouse model. Int J Mol Sci (2022) 23:12672. doi: 10.3390/ijms232012672 36293529 PMC9603933

[91] Yang L Wu G Wu Q Peng L Yuan L. METTL3 overexpression aggravates LPS-induced cellular inflammation in mouse intestinal epithelial cells and DSS-induced IBD in mice. Cell Death Discovery (2022) 8:62. doi: 10.1038/s41420-022-00849-1 35165276 PMC8844074

[92] Shen Z-J Han Y-C Nie M-W Wang Y-N Xiang R-L Xie H-Z. Genome-wide identification of altered RNA m(6)A profiles in vascular tissue of septic rats. Aging (2021) 13:21610–27. doi: 10.18632/aging.203506 PMC8457599 34507301

[93] Zhang H Liu J Zhou Y Qu M Wang Y Guo K. Neutrophil extracellular traps mediate m(6)A modification and regulates sepsis-associated acute lung injury by activating ferroptosis in alveolar epithelial cells. Int J Biol Sci (2022) 18:3337–57. doi: 10.7150/ijbs.69141 PMC9134924 35637949

[94] Qu M Chen Z Qiu Z Nan K Wang Y Shi Y. Neutrophil extracellular traps-triggered impaired autophagic flux via METTL3 underlies sepsis-associated acute lung injury. Cell Death Discovery (2022) 8:375. doi: 10.1038/s41420-022-01166-3 36030287 PMC9420153

[95] Chen Y Wu Y Zhu L Chen C Xu S Tang D. METTL3-mediated N6-methyladenosine modification of trim59 mRNA protects against sepsis-induced acute respiratory distress syndrome. Front Immunol (2022) 13:897487. doi: 10.3389/fimmu.2022.897487 35693774 PMC9174697

[96] Zhao D Wang C Liu X Liu N Zhuang S Zhang Q. CircN4bp1 Facilitates Sepsis-Induced Acute Respiratory Distress Syndrome through Mediating Macrophage Polarization via the miR-138-5p/EZH2 Axis. Mediators Inflammation (2021) 2021:7858746. doi: 10.1155/2021/7858746 PMC8739551 35002536

[97] Yang Y Yang X Wu Y Fu M. METTL3 promotes inflammation and cell apoptosis in a pediatric pneumonia model by regulating EZH2. Allergol Immunopathol (Madr) (2021) 49:49–56. doi: 10.15586/aei.v49i5.445 34476922

[98] Li S-X Yan W Liu J-P Zhao Y-J Chen L. Long noncoding RNA SNHG4 remits lipopolysaccharide-engendered inflammatory lung damage by inhibiting METTL3 - Mediated m(6)A level of STAT2 mRNA. Mol Immunol (2021) 139:10–22. doi: 10.1016/j.molimm.2021.08.008 34450538

[99] Li N Hui H Bray B Gonzalez GM Zeller M Anderson KG. METTL3 regulates viral m6A RNA modification and host cell innate immune responses during SARS-CoV-2 infection. Cell Rep (2021) 35:109091. doi: 10.1016/j.celrep.2021.109091 33961823 PMC8090989

[100] Huang L Tang H Hu J. METTL3 Attenuates Inflammation in Fusarium solani-Induced Keratitis via the PI3K/AKT Signaling Pathway. Invest Ophthalmol Vis Sci (2022) 63:20. doi: 10.1167/iovs.63.10.20 PMC9526359 36169946

[101] Tang H Huang L Hu J. Inhibition of the m6A Methyltransferase METTL3 Attenuates the Inflammatory Response in Fusarium solani-Induced Keratitis via the NF-κB Signaling Pathway. Invest Ophthalmol Vis Sci (2022) 63:2. doi: 10.1167/iovs.63.11.2 PMC9547362 36194423

[102] Meng J Liu X Tang S Liu Y Zhao C Zhou Q. METTL3 inhibits inflammation of retinal pigment epithelium cells by regulating NR2F1 in an m(6)A-dependent manner. Front Immunol (2022) 13:905211. doi: 10.3389/fimmu.2022.905211 35936005 PMC9351451

[103] Feng Z Li Q Meng R Yi B Xu Q. METTL3 regulates alternative splicing of MyD88 upon the lipopolysaccharide-induced inflammatory response in human dental pulp cells. J Cell Mol Med (2018) 22:2558–68. doi: 10.1111/jcmm.13491 PMC5908103 29502358

[104] Yin H Zhang X Yang P Zhang X Peng Y Li D. RNA m6A methylation orchestrates cancer growth and metastasis via macrophage reprogramming. Nat Commun (2021) 12:1394. doi: 10.1038/s41467-021-21514-8 33654093 PMC7925544

[105] He J Zhou M Yin J Wan J Chu J Jia J. METTL3 restrains papillary thyroid cancer progression via m(6)A/c-Rel/IL-8-mediated neutrophil infiltration. Mol Ther J Am Soc Gene Ther (2021) 29:1821–37. doi: 10.1016/j.ymthe.2021.01.019 PMC8116572 33484966

[106] Jia G Wang X Wu W Zhang Y Chen S Zhao J. LXA4 enhances prostate cancer progression by facilitating M2 macrophage polarization via inhibition of METTL3. Int Immunopharmacol (2022) 107:108586. doi: 10.1016/j.intimp.2022.108586 35228032

[107] Wu L Cheng D Yang X Zhao W Fang C Chen R. M2-TAMs promote immunoresistance in lung adenocarcinoma by enhancing METTL3-mediated m6A methylation. Ann Transl Med (2022) 10:1380. doi: 10.21037/atm-22-6104 36660648 PMC9843413

[108] Lan H Liu Y Liu J Wang X Guan Z Du J. Tumor-Associated Macrophages Promote Oxaliplatin Resistance via METTL3-Mediated m(6)A of TRAF5 and Necroptosis in Colorectal Cancer. Mol Pharm (2021) 18:1026–37. doi: 10.1021/acs.molpharmaceut.0c00961 33555197

[109] Wang A Tao W Tong J Gao J Wang J Hou G. m6A modifications regulate intestinal immunity and rotavirus infection. eLife (2022) 11:e73628. doi: 10.7554/eLife.73628 35098923 PMC8860440

[110] Zhong C Tao B Yang F Xia K Yang X Chen L. Histone demethylase JMJD1C promotes the polarization of M1 macrophages to prevent glioma by upregulating miR-302a. Clin Transl Med (2021) 11:e424. doi: 10.1002/ctm2.424 34586733 PMC8473479

[111] Falk E. Pathogenesis of atherosclerosis. J Am Coll Cardiol (2006) 47:C7–12. doi: 10.1016/j.jacc.2005.09.068 16631513

[112] Powell EE Wong VW-S Rinella M. Non-alcoholic fatty liver disease. Lancet Lond Engl (2021) 397:2212–24. doi: 10.1016/S0140-6736(20)32511-3 33894145

[113] Barajas JM Lin C-H Sun H-L Alencastro F Zhu AC Aljuhani M. METTL3 regulates liver homeostasis, hepatocyte ploidy, and circadian rhythm-controlled gene expression in mice. Am J Pathol (2022) 192:56–71. doi: 10.1016/j.ajpath.2021.09.005 34599880 PMC8759040

[114] Maiuolo J Oppedisano F Gratteri S Muscoli C Mollace V. Regulation of uric acid metabolism and excretion. Int J Cardiol (2016) 213:8–14. doi: 10.1016/j.ijcard.2015.08.109 26316329

[115] Zhang S Wang Y Cheng J Huangfu N Zhao R Xu Z. HyPeruricemia and cardiovascular disease. Curr Pharm Des (2019) 25:700–9. doi: 10.2174/1381612825666190408122557 30961478

[116] Afsar B Elsurer R Covic A Johnson RJ Kanbay M. Relationship between uric acid and subtle cognitive dysfunction in chronic kidney disease. Am J Nephrol (2011) 34:49–54. doi: 10.1159/000329097 21659739 PMC3121541

[117] Tian T Liu X Li T Nie Z Li S Tang Y. Detrimental effects of long-term elevated serum uric acid on cognitive function in rats. Sci Rep (2021) 11:6732. doi: 10.1038/s41598-021-86279-y 33762656 PMC7991666

[118] Zhou Y Zhao M Pu Z Xu G Li X. Relationship between oxidative stress and inflammation in hyPeruricemia: Analysis based on asymptomatic young patients with primary hyPeruricemia. Med (Baltimore) (2018) 97:e13108. doi: 10.1097/MD.0000000000013108 PMC6310523 30544373

[119] Galicia-Garcia U Benito-Vicente A Jebari S Larrea-Sebal A Siddiqi H Uribe KB. Pathophysiology of type 2 diabetes mellitus. Int J Mol Sci (2020) 21:6275. doi: 10.3390/ijms21176275 32872570 PMC7503727

[120] Kato M Natarajan R. Epigenetics and epigenomics in diabetic kidney disease and metabolic memory. Nat Rev Nephrol (2019) 15:327–45. doi: 10.1038/s41581-019-0135-6 PMC6889804 30894700

[121] Guilak F Nims RJ Dicks A Wu C-L Meulenbelt I. Osteoarthritis as a disease of the cartilage pericellular matrix. Matrix Biol J Int Soc Matrix Biol (2018) 71–72:40–50. doi: 10.1016/j.matbio.2018.05.008 PMC6146061 29800616

[122] Risbud MV Shapiro IM. Role of cytokines in intervertebral disc degeneration: pain and disc content. Nat Rev Rheumatol (2014) 10:44–56. doi: 10.1038/nrrheum.2013.160 24166242 PMC4151534

[123] Francisco V Pino J González-Gay MÁ Lago F Karppinen J Tervonen O. A new immunometabolic perspective of intervertebral disc degeneration. Nat Rev Rheumatol (2022) 18:47–60. doi: 10.1038/s41584-021-00713-z 34845360

[124] Lieben L Marshall L. Rheumatoid arthritis. Nat Rev Dis Primer (2018) 4:18002. doi: 10.1038/nrdp.2018.2 29417950

[125] McInnes IB Schett G. The pathogenesis of rheumatoid arthritis. N Engl J Med (2011) 365:2205–19. doi: 10.1056/NEJMra1004965 22150039

[126] Baxter D McInnes IB Kurowska-Stolarska M. Novel regulatory mechanisms in inflammatory arthritis: a role for microRNA. Immunol Cell Biol (2012) 90:288–92. doi: 10.1038/icb.2011.114 22249200

[127] Klein K Gay S. Epigenetics in rheumatoid arthritis. Curr Opin Rheumatol (2015) 27:76–82. doi: 10.1097/BOR.0000000000000128 25415526

[128] Griffiths CEM Armstrong AW Gudjonsson JE Barker JNWN. Psoriasis. Lancet Lond Engl (2021) 397:1301–15. doi: 10.1016/S0140-6736(20)32549-6 33812489

[129] Boehncke W-H Schön MP. Psoriasis. Lancet Lond Engl (2015) 386:983–94. doi: 10.1016/S0140-6736(14)61909-7 26025581

[130] Kagami S Rizzo HL Lee JJ Koguchi Y Blauvelt A. Circulating th17, th22, and th1 cells are increased in psoriasis. J Invest Dermatol (2010) 130:1373–83. doi: 10.1038/jid.2009.399 PMC2892169 20032993

[131] Gutcher I Becher B. APC-derived cytokines and T cell polarization in autoimmune inflammation. J Clin Invest (2007) 117:1119–27. doi: 10.1172/JCI31720 PMC1857272 17476341

[132] Chen C-J Hu H Liao W-T. Pathophysiology of inflammatory bowel diseases. N Engl J Med (2021) 384:1376–7. doi: 10.1056/NEJMc2101562 33826830

[133] Sebastian-delaCruz M Olazagoitia-Garmendia A Gonzalez-Moro I Santin I Garcia-Etxebarria K Castellanos-Rubio A. Implication of m6A mRNA Methylation in Susceptibility to Inflammatory Bowel Disease. Epigenomes (2020) 4:16. doi: 10.3390/epigenomes4030016 34968289 PMC8594712

[134] Singer M Deutschman CS Seymour CW Shankar-Hari M Annane D Bauer M. The third international consensus definitions for sepsis and septic shock (Sepsis-3). JAMA (2016) 315:801–10. doi: 10.1001/jama.2016.0287 PMC4968574 26903338

[135] Vincent J-L Sakr Y Sprung CL Ranieri VM Reinhart K Gerlach H. Sepsis in European intensive care units: Results of the SOAP study*. Crit Care Med (2006) 34:344–53. doi: 10.1097/01.ccm.0000194725.48928.3a 16424713

[136] Karlsson S Varpula M Ruokonen E Pettilä V Parviainen I Ala-Kokko TI. Incidence, treatment, and outcome of severe sepsis in ICU-treated adults in Finland: the Finnsepsis study. Intensive Care Med (2007) 33:435–43. doi: 10.1007/s00134-006-0504-z 17225161

[137] Hotchkiss RS Karl IE. The pathophysiology and treatment of sepsis. N Engl J Med (2003) 348:138–50. doi: 10.1056/NEJMra021333 12519925

[138] Cecconi M Evans L Levy M Rhodes A. Sepsis and septic shock. Lancet (2018) 392:75–87. doi: 10.1016/S0140-6736(18)30696-2 29937192

[139] Torres A Cilloniz C Niederman MS Menéndez R Chalmers JD Wunderink RG. Pneumonia. Nat Rev Dis Primer (2021) 7:25. doi: 10.1038/s41572-021-00259-0 33833230

[140] Xu Q Tang Y Huang G. Innate immune responses in RNA viral infection. Front Med (2021) 15:333–46. doi: 10.1007/s11684-020-0776-7 PMC7862985 33263837

[141] Zheng X Wang J Zhang X Fu Y Peng Q Lu J. RNA m(6) A methylation regulates virus-host interaction and EBNA2 expression during Epstein-Barr virus infection. Immun Inflammation Dis (2021) 9:351–62. doi: 10.1002/iid3.396 PMC8127537 33434416

[142] Brown L Leck AK Gichangi M Burton MJ Denning DW. The global incidence and diagnosis of fungal keratitis. Lancet Infect Dis (2021) 21:e49–57. doi: 10.1016/S1473-3099(20)30448-5 33645500

[143] Shacter E Weitzman SA. Chronic inflammation and cancer. Oncol Williston Park N (2002) 16:217–226, 229. 11866137

[144] Wang B Liu Y Jiang R Liu Z Gao H Chen F. Emodin relieves the inflammation and pyroptosis of lipopolysaccharide-treated 1321N1 cells by regulating methyltransferase-like 3 -mediated NLR family pyrin domain containing 3 expression. Bioengineered (2022) 13:6740–9. doi: 10.1080/21655979.2022.2045836 PMC8973593 35246004

[145] Xu R Xiao X Zhang S Pan J Tang Y Zhou W. The methyltransferase METTL3-mediated fatty acid metabolism revealed the mechanism of cinnamaldehyde on alleviating steatosis. BioMed Pharmacother Biomed Pharmacother (2022) 153:113367. doi: 10.1016/j.biopha.2022.113367 35780619

[146] Izquierdo V Palomera-Ávalos V Pallàs M Griñán-Ferré C. Resveratrol Supplementation Attenuates Cognitive and Molecular Alterations under Maternal High-Fat Diet Intake: Epigenetic Inheritance over Generations. Int J Mol Sci (2021) 22:1453. doi: 10.3390/ijms22031453 33535619 PMC7867164

[147] Wu J Li Y Yu J Gan Z Wei W Wang C. Resveratrol attenuates high-fat diet induced hepatic lipid homeostasis disorder and decreases m6A RNA methylation. Front Pharmacol (2020) 11:568006. doi: 10.3389/fphar.2020.568006 33519432 PMC7845411

[148] Zhou H Shen X Yan C Xiong W Ma Z Tan Z. Extracellular vesicles derived from human umbilical cord mesenchymal stem cells alleviate osteoarthritis of the knee in mice model by interacting with METTL3 to reduce m6A of NLRP3 in macrophage. Stem Cell Res Ther (2022) 13:322. doi: 10.1186/s13287-022-03005-9 35842714 PMC9288728

[149] Dong X Fu J Yin X Cao S Li X Lin L. Emodin: A review of its pharmacology, toxicity and pharmacokinetics. Phytother Res PTR (2016) 30:1207–18. doi: 10.1002/ptr.5631 PMC7168079 27188216

[150] Neto JGO Boechat SK Romão JS Pazos-Moura CC Oliveira KJ. Treatment with cinnamaldehyde reduces the visceral adiposity and regulates lipid metabolism, autophagy and endoplasmic reticulum stress in the liver of a rat model of early obesity. J Nutr Biochem (2020) 77:108321. doi: 10.1016/j.jnutbio.2019.108321 31869758

[151] Li N Tang H Wu L Ge H Wang Y Yu H. Chemical constituents, clinical efficacy and molecular mechanisms of the ethanol extract of Abelmoschus manihot flowers in treatment of kidney diseases. Phytother Res (2021) 35:198–206. doi: 10.1002/ptr.6818 32716080 PMC7891592

[152] Li J Zhang C-X Liu Y-M Chen K-L Chen G. A comparative study of anti-aging properties and mechanism: resveratrol and caloric restriction. Oncotarget (2017) 8:65717–29. doi: 10.18632/oncotarget.20084 PMC5630366 29029466

[153] Lange KW. Red wine, resveratrol, and Alzheimer’s disease. Mov Nutr Health Dis (2018) 2:31–8. doi: 10.5283/mnhd.11

